# The Peripheral(-Muscle) Oxygenation and Perfusion Score (POP-Score): A New Non-Invasive Tool Associated with Elevations in C-Reactive Protein Levels in Neonates

**DOI:** 10.3390/diagnostics16101447

**Published:** 2026-05-09

**Authors:** Christina H. Wolfsberger, Christoph Schlatzer, Ena Suppan, Marlies Bruckner, Nina Hoeller, Bernhard Schwaberger, Gerhard Pichler

**Affiliations:** 1 Division of Neonatology, Department of Paediatrics and Adolescent Medicine, Medical University of Graz, 8036 Graz, Austria; 2 Research Unit for Neonatal Micro- and Macrocirculation, Department of Paediatrics and Adolescent Medicine, Medical University of Graz, 8036 Graz, Austria; 3 Research Unit for Cerebral Development and Oximetry Research, Medical University of Graz, 8036 Graz, Austria

**Keywords:** peripheral-muscle oxygenation, NIRS, infection, predictive values, infection score, neonate, POP-Score

## Abstract

**Background/Objectives**: Peripheral(-muscle) oxygenation assessed with near-infrared spectroscopy might serve as an early marker of infection/inflammation; however, evidence of its clinical relevance is lacking so far. This study aimed to develop a peripheral(-muscle) oxygenation and perfusion score (POP-Score) using the peripheral(-muscle) tissue oxygenation index (pTOI) combined with non-invasive monitoring parameters within six hours after birth. The POP-Score was designed to explore associations with elevated C-reactive protein (CRP), as an early infection/inflammation marker, in term and late-preterm neonates. **Methods**: Secondary outcome parameters from a prospective observational study were analysed. Included neonates weighed ≥2000 g with respiratory distress, excluding those with umbilical artery pH < 7.20. Neonates with CRP ≥ 20 mg/L were 1:4-matched to those with CRP < 20 mg/L by gestational age (±2 weeks). For pTOI measurements, a sensor was placed for a duration of 30 s, followed by four further reapplications of the sensor, using the NIRO200NX within the first six hours after birth. The POP-Score was established using the following formula: (*pTOI* [%] × *subcutaneous fat layer thickness* [cm] × *heart rate* [bpm])/(*arterial oxygen saturation* [%] × *systolic blood pressure* [mmHg]). POP-Score was correlated with the highest CRP within 48 h. **Results**: Thirty neonates were included (median gestational age: 39.1 weeks [CRP < 20 mg/L group] vs. 37.3 weeks [CRP ≥ 20 mg/L group], *p* = 0.299; median birth weight: 3561 g vs. 3260 g, *p* = 0.058, respectively). Median POP-Scores were significantly different: 1.11 (CRP ≥ 20 mg/L) vs. 0.85 (CRP < 20 mg/L), *p* < 0.001. POP-Score correlated positively with CRP (r = 0.341; *p* = 0.070). In this small exploratory cohort, a POP-Score cut-off of 1.00 was associated with CRP ≥ 20 mg/L (100% sensitivity and 87% specificity); however, these estimates are uncertain due to the limited sample size. **Conclusions**: This study is the first to describe a new score for peripheral(-muscle) oxygenation and perfusion (POP-Score), which may represent a potential approach for early, non-invasive assessment but requires validation in adequately powered studies before any clinical application. **Trial Registration**: Clinicaltrials.gov, Trial registration number: NCT04818762, Date of Registration: 26 March 2021.

## 1. Introduction

Neonatal infections are among the most common causes of admissions to the neonatal intensive care units (NICUs) worldwide, with a higher prevalence in low-income countries [1,2]. Within the first hours after birth, symptoms of neonatal infections, primarily respiratory distress, can be subtle and difficult to distinguish from symptoms associated with infant respiratory distress syndrome due to prematurity or transient respiratory distress of the newborn [3]. Diagnosing the underlying disease is challenging and requires interpretation of clinical signs in combination with risk factors [4,5,6,7] and laboratory findings. Infection parameters can be analysed from cord or newborn blood after birth [6,8,9]. However, parameters obtained from the newborn may be influenced by various factors other than infection, such as asphyxia or gestational- and postnatal age [4]. The gold standard for the diagnosis of infection/sepsis is a positive blood culture [6,10]. However, sampling blood cultures in neonates, particularly in preterm neonates, is not always feasible in clinical practice due to the required blood volume [8]. Insufficient blood volume (0.5 mL) is linked to false-negative results, and contamination can cause false-positive results [4,11]. Consequently, laboratory parameters are often used in clinical practice to diagnose bacterial infection and initiate antibiotic therapy. Leukocyte counts outside the normal range (leukopenia < 6000/µL or leukocytosis > 30,000/µL) within the first days after birth have high predictive values for sepsis [12]. The immature-total neutrophil ratio (IT ratio), with a cut-off parameter of greater than 0.2, has a high sensitivity for predicting infections [12]. C-reactive protein (CRP) levels are often used to diagnose neonatal sepsis, but their accuracy within the first 48 h of life is debated. Confounding factors such as delivery type and hypoxia can significantly influence CRP values, potentially limiting their reliability as an early predictor of sepsis [13,14]. Additionally, C-reactive protein is of considerable importance, although its rise in cases of infections is often delayed by ten to twelve hours, complicating early diagnosis of neonatal infections [15]. Beside the previously mentioned limitations of blood parameters, the sampling procedures are invasive. Thus, a non-invasive approach in neonatal care could be advantageous for reducing the need for painful procedures.

In recent decades, measurements of peripheral(-muscle) oxygenation using near-infrared spectroscopy (NIRS) have attracted increasing interest as a non-invasive method [16,17,18,19,20,21,22]. Normal values have already been described in stable neonates [23,24,25]. Using peripheral(-muscle) NIRS measurements, information about peripheral(-muscle) tissue oxygenation (pTOI) and peripheral(-muscle) fractional tissue oxygen extraction (pFTOE)—when arterial oxygen saturation (SpO_2_) is measured simultaneously using pulse oximetry—can be obtained [22,26,27].

A potential association between leukocyte counts [28] and CRP with peripheral(-muscle) oxygenation [17] and cerebral oxygenation [29], measured by NIRS, has already been described. These associations raise the question of whether peripheral(-muscle) oxygenation can contribute to the early detection of microcirculatory disorders in order to identify neonates at risk of sepsis/infection before other clinical signs or laboratory findings appear. To enhance the interpretation of peripheral(-muscle) oxygenation in early inflammatory stages in preterm neonates, it seems necessary to combine NIRS with other influencing parameters [18].

Therefore, we aimed to define a score using pTOI in combination with other non-invasively measurable parameters within the first six hours after birth. This tool, termed peripheral(-muscle) oxygenation and perfusion score (POP-Score) aims to predict elevated CRP levels as an early potential indicator of infection/inflammation in term and late-preterm neonates. We hypothesised that a cut-off value of the generated POP-Score would have a high sensitivity and specificity for the prediction of elevated CRP levels.

## 2. Materials and Methods

### 2.1. Study Design

The present study analysed secondary outcome parameters from a prospective observational study, the pFTOE trial [24] conducted between March 2021 and March 2024 at the NICU of the Division of Neonatology, Medical University of Graz. The original study was reviewed and approved by the local Committee on Biomedical Research Ethics (EC-Number: 33–161 ex 20/21). Informed consent was obtained from the parents after birth prior to inclusion in the study. The pFTOE trial was registered at Clinical Trials.gov (NCT04818762, 26 March 2021). The study was performed in line with the principles of the Declaration of Helsinki.

### 2.2. Patients

Term and preterm neonates with a gestational age of ≥30 weeks exhibiting signs of respiratory distress (need for respiratory support and/or supplemental oxygen, retractions, nasal flaring, grunting, tachypnoea) and risk factors for infection (premature rupture of membranes > 16 h before birth, maternal signs of infection during labour [fever, elevated inflammatory markers], or amniotic fluid infection) and admitted to the NICU Graz were eligible for inclusion in the *pFTOE trial*. Neonates with severe congenital malformations were excluded. For the present analysis, neonates with a birth weight ≥ 2000 g, an umbilical artery pH > 7.20, and measurements of peripheral(-muscle) oxygenation within six hours after birth were included. Therefore, neonates with a birth weight below 2000 g or an umbilical artery pH less than 7.20 were excluded from the analysis in order to establish a relatively uniform study population for the development of the POP-Score. Neonates with CRP ≥ 20 mg/L were matched in a ratio of 1:4 to those neonates with CRP < 20 mg/L, matching for gestational age within ± two weeks to minimise potential bias from differences in gestational age.

### 2.3. Procedure

Medical history and demographic data including gestational age, birth weight, gender, Apgar scores, blood gas analysis from the umbilical artery, postnatal blood samples, mode of delivery, and maternal perinatal history were documented for each neonate. To detect a possible infection, elevated leukocyte counts, CRP levels, and IT ratio were documented within the first 48 h after birth. The subcutaneous fat layer thickness of the right forearm was evaluated by ultrasound using the Logiq S8 (GE Health Care, Chicago, IL, USA).

### 2.4. Non-Invasive Monitoring

Routine non-invasive monitoring included pulse oximetry on the right forearm/wrist for preductal SpO_2_ measurement, electrocardiography for heart rate (HR) measurement, and non-invasive blood pressure measurement performed using IntelliVue MP70 or MX750 monitor (Philips, Eindhoven, The Netherlands). Central body temperature measurements were performed with a rectal probe.

Peripheral(-muscle) oxygenation measurements were performed once within the first 6 h after birth, following a rest period of at least 15 min for the neonate. For pTOI measurements, the NIRO 200 NX (Hamamatsu Photonics K.K, Hamamatsu City, Japan) was used, with the neonatal NIRS sensor attached to the right forearm using an interoptode distance of 3.0 cm.

The sensor was held in position gently without applying pressure while shielding the light. When stable signals were obtained for approximately 30 s, the sensor was removed for a 10-s rest period, followed by four further reapplications of the sensor at approximately the same position. A total of five measurements were carried out. The mean of the five pTOI measurements was calculated. pFTOE was calculated by using the following formula: (SpO_2_-pTOI)/SpO_2_. SpO_2_ and HR were recorded continuously, and the mean was calculated during the peripheral(-muscle) NIRS measurements.

To minimize NIRS measurement variability, a standardized protocol was applied. The NIRS sensor was consistently placed on the right forearm at the same anatomical location. Care was taken to avoid excessive pressure and to shield the sensor from ambient light. Measurements were performed during a resting state of the neonate to reduce motion artefacts. To improve reliability and reproducibility, five consecutive measurements were obtained with short repositioning intervals, and the mean value was used for further analysis.

Immediately before and after the peripheral(-muscle) NIRS measurements, arterial blood pressure was assessed—including systolic blood pressure (SABP), diastolic blood pressure (DABP), and mean arterial blood pressure (MABP)—using a pneumatic cuff.

### 2.5. Peripheral(-Muscle) Oxygenation and Perfusion Score (POP-Score)

**Figure 1:**
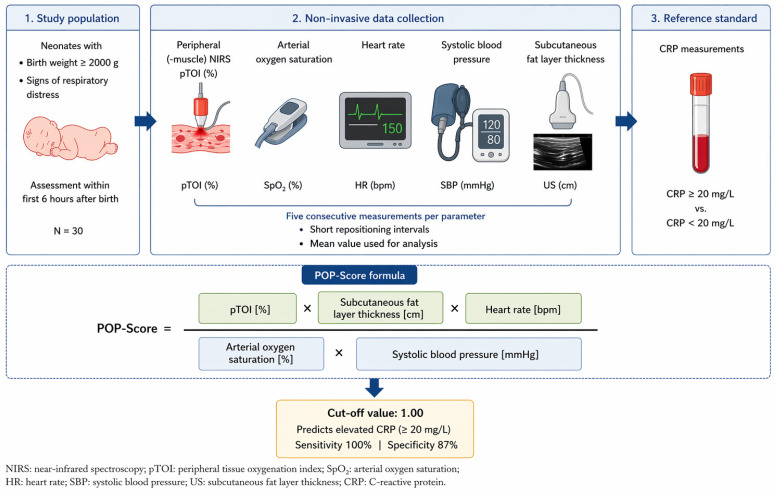
Derivation of the POP-Score: study design, data collection, and score calculation.

A non-invasive infection score was established using pTOI, measured by peripheral(-muscle) NIRS, combined with SpO_2_, HR, SABP, and subcutaneous fat layer thickness (Figure 1). The mean of five pTOI measurements contributed to the formula. SpO_2_ and HR were recorded continuously, and the mean was calculated during the peripheral(-muscle) NIRS measurements and contributed to the POP-Score. The mean SABP measurement before and after peripheral(-muscle) NIRS measurements was used and contributed to the POP-Score. The subcutaneous fat layer thickness of the right forearm was evaluated by ultrasound and also contributed to the POP-Score.

$$POP\text{-}Score=\frac{pTOI\;\left[\%\right]\times subcutaneous\;fat\;layer\;thickness\;\left[cm\right]\times heart\;rate\;[bpm]}{arterial\;oxygen\;saturation\;\left[\%\right]\times systolic\;blood\;pressure\;[mmHg]}$$

The POP-Score was developed as a hypothesis-generating tool based on clinical reasoning and previously described associations between peripheral(-muscle) oxygenation and routinely monitored parameters. Due to the limited sample size and the small number of neonates with elevated CRP levels, no regression analysis or other data-driven modelling approaches were performed. Consequently, no statistically derived weighting or coefficients were applied to individual components of the score. All variables were incorporated into the POP-Score formula in a non-weighted manner, reflecting their presumed physiological relevance. Internal validation (e.g., bootstrapping or split-sample validation) was not performed due to the limited sample size.

### 2.6. Infectious Variables

In included neonates with respiratory distress and risk factors for infection who were admitted to the NICU, blood samples for laboratory investigations on the first and second day after birth were routinely performed. Infection parameters included leukocyte counts, CRP levels and IT ratio. The highest values of leukocyte counts, CRP and IT ratio obtained within the first 48 h after birth were used for further analyses.

### 2.7. Statistical Analysis

Demographic data were described as median and minimum/maximum for continuous variables. Categorical data were noted as absolute and relative numbers. Baseline differences between groups were analysed using a *t*-test or Mann–Whitney U test for continuous data and Chi-square test or Fisher’s exact test for categorical data. A correlation analysis between CRP levels and POP-Score was carried out using Spearman’s rank correlation for non-normally distributed data. A *p*-value < 0.05 was considered statistically significant. The sensitivity and specificity of the cut-off value of the POP-Score were calculated to predict a CRP level ≥ 20 mg/L. Sub-analyses were performed for neonates below and above the cut-off value. Due to the limited sample size and exploratory nature of the study, no receiver operating characteristic (ROC) analysis or model calibration assessment was performed. Statistical analyses were performed using SPSS Statistics 29 (IBM Corporation, Armonk, NY, USA).

## 3. Results

**Figure 2:**
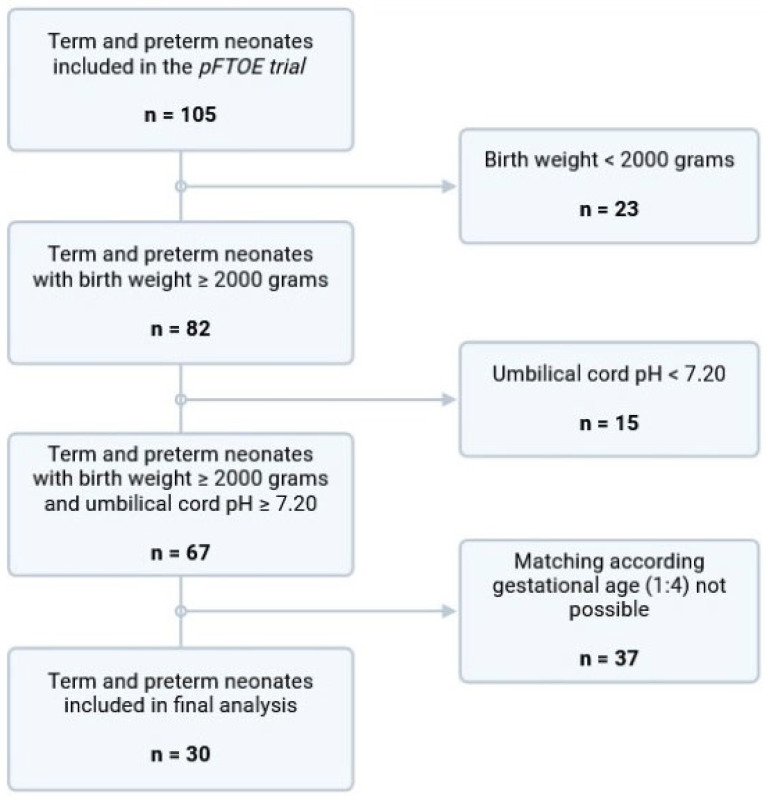
Study flow chart of included term and late-preterm neonates.

A total of 105 neonates > 30 weeks of gestation were included in the prospective observational study obtaining peripheral(-muscle) NIRS measurements within the first six hours after birth. Twenty-three neonates were excluded due to a birth weight < 2000 g. Six neonates had elevated CRP levels ≥ 20 mg/L. These neonates were matched with 24 neonates with CRP levels < 20 mg/L. Some 37 neonates were excluded, as matching by gestational age was not possible. Thus, a total of 30 neonates were included in the final analysis (Figure 2).

### 3.1. Demographic Data

The median (minimum; maximum) gestational ages of the neonates in the CRP ≥ 20 mg/L and CRP < 20 mg/L groups were 39.1 (36.6; 41.3) and 37.3 (36.1; 41.9) weeks (*p* = 0.299), respectively. The median (minimum; maximum) birth weights in the CRP ≥ 20 mg/L and CRP < 20 mg/L groups were 3561 (2985; 4360) and 3260 (2120; 3950) grams (*p* = 0.058), respectively.

Demographic data, routine monitoring parameters and peripheral(-muscle) NIRS data are displayed in Table 1. Blood gas analyses were performed at intervals of median (minimum; maximum) 39 (30; 85) minutes to peripheral(-muscle) NIRS measurement in neonates with CRP ≥ 20 mg/L and 36 (13; 95) minutes in neonates with CRP < 20 mg/L. In the CRP ≥ 20 mg/L group, four neonates (66.7%) were delivered by primary Cesarean section and two (33.3%) by secondary Cesarean section. In the CRP < 20 mg/L group, 21 (87.5%) neonates were delivered by primary Cesarean section, 1 (4.2%) by secondary Cesarean section, and 2 (8.3%) delivered vaginally.

### 3.2. POP-Score

**Figure 3:**
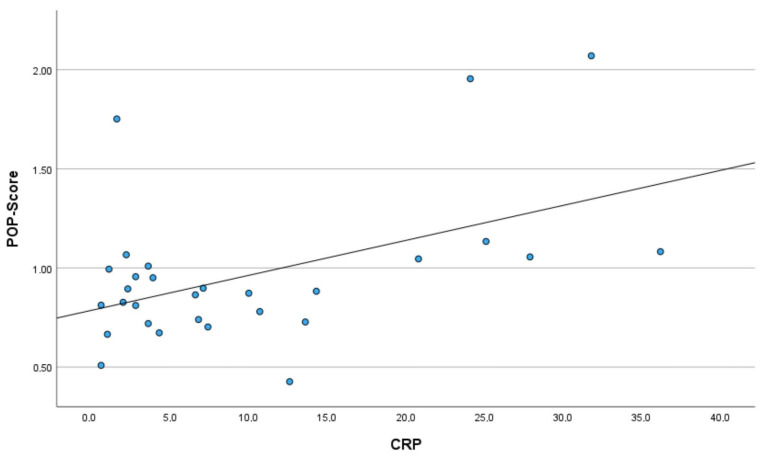
Correlation of the peripheral(-muscle) oxygenation and perfusion score (POP-Score) with C-reactive protein (CRP; mg/L) in 30 included term and late-preterm neonates. Each dot represents one patient.

All parameters contributing to POP-Score, including parameters of peripheral(-muscle) oxygenation and routine monitoring parameters, are displayed in Table 1. The median (minimum; maximum) POP-Score was 1.11 (1.05; 1.96) in neonates with CRP ≥ 20 mg/L and 0.85 (0.43; 1.75) (*p* < 0.001) in those with CRP < 20 mg/L, respectively. The median (minimum; maximum) CRP was 24.6 (20.8; 36.2) mg/L in neonates stratified to CRP ≥ 20 mg/L and 5.1 (1.0; 14.3) mg/L in neonates with CRP < 20 mg/L. All neonates with elevated CRP levels ≥ 20 mg/L had a POP-Score ≥ 1.00. This POP-Score cut-off value of 1.00 had a sensitivity of 100% and specificity of 87% for being associated with CRP levels ≥ 20 mg/L. The POP-Score tended to correlate positively with CRP (r = 0.341; *p* = 0.070) (Figure 3). No pathogen identification was achieved in the overall cohort. Blood cultures were obtained in 3 of 6 neonates (50%) in the CRP ≥ 20 mg/L group and in 20 of 24 neonates (83%) in the CRP < 20 mg/L group; however, all cultures remained negative.

## 4. Discussion

In the present study we successfully established a score (POP-Score) for exploring associations with elevated CRP levels ≥ 20 mg/L in term and late-preterm neonates. By defining a cut-off value of 1.00 with a sensitivity of 100% and specificity of 87%, this score showed an association with elevated CRP levels in this exploratory dataset. The POP-Score comprises various non-invasively measured parameters such as peripheral(-muscle) NIRS measurements (pTOI), routine monitoring parameters (SpO_2_, HR, SABP), and sonographic assessment of composition of the measured tissue (subcutaneous fat layer thickness of the forearm). Additionally, we demonstrated a trend towards a positive correlation between POP-Score and CRP levels.

A critical aspect of this study is the use of CRP as a surrogate marker. As CRP is a non-specific inflammatory parameter and may be elevated in non-infectious conditions, the POP-Score should be interpreted as reflecting inflammatory response rather than confirmed infection [14]. In this context, the POP-Score should be interpreted as a potential indicator of inflammatory response rather than confirmed infection, and further validation using clinical sepsis definitions is required.

Previous studies have investigated the benefit of peripheral(-muscle) NIRS in different clinical situations, including infection, anaemia and arterial hypotension, focusing on selected single parameters [16,17,19,20,28]. The potential association between peripheral(-muscle) NIRS measurements and CRP levels has already been described [17]. They compared peripheral(-muscle) oxygenation and perfusion parameters in neonates with elevated CRP levels > 10 mg/L to those without CRP level elevation. Their findings indicated that peripheral(-muscle) oxygenation and perfusion were reduced in neonates with elevated CRP levels > 10 mg/L, while routine monitoring parameters, such as SpO_2_, HR, or MABP, remained unaffected. In addition to CRP, Binder et al. investigated the potential influence of leukocyte counts on peripheral(-muscle) oxygenation, again in combination with the venous occlusion method [28]. They demonstrated a negative correlation of peripheral(-muscle) oxygen consumption with increasing leukocyte counts.

Up to now, the implementation of peripheral(-muscle) NIRS measurements in clinical routine, particularly in situations of sepsis/infection, has been hindered by the wide variations in observed values that complicate individual decisions. Our results suggest that the use of a score combining non-invasively measured parameters (pTOI, HR, SpO_2_, SABP, subcutaneous fat layer thickness) and a predefined cut-off value serve as a potential indicator for the detection of infection at early stages.

The potential clinical value of the POP-Score lies in providing early, non-invasive physiological information within the first hours after birth. It is intended to complement, not replace, established diagnostic approaches.

SpO_2_ was included in the formula because a strong correlation with pTOI had already been described previously [18]. To account for SpO_2_, pTOI was divided by SpO_2_ to establish a ratio. Pichler et al. [18] described a significant positive correlation between SpO_2_ and pTOI, noting that in early stages of infection, SpO_2_ typically remains within the normal range [4,30]. Neonates diagnosed with early stages of sepsis/infection usually present with an increased apnoea rate, but a decrease in SpO_2_ is uncommon [30].

Haemodynamic parameters, including HR and blood pressure, were included in the formula due to their common changes during infections. Changes in HR include a decline in HR variability, persistent tachycardia or, more frequently, repetitive or transient bradycardia [30]. In stable neonates, HR is negatively associated with pTOI [18]. Since bradycardia is often observed during infections, HR was multiplied by pTOI to account for potential perfusion disturbances.

Arterial hypotension resulting from a decrease in cardiac preload or changes in systemic vascular resistance occurs in neonatal sepsis [30]. During sepsis/infection, SABP is more informative than MABP in cases of reduced left ventricular output [30]. Furthermore, SABP and DABP show a more pronounced decrease in situations of warm shock compared to MABP [31]. The literature has described an association/correlation between MABP and pTOI [18]. Due to its potential for detecting early disturbances in the cardiovascular system, SABP was included in the present formula. Since SABP may indicate early situations of shock in infected neonates, pTOI was divided by SABP to account for negative correlation with pTOI.

The composition of the tissue, primarily subcutaneous fat, significantly influences pTOI [18]. Subcutaneous tissue is positively associated with pTOI in stable neonates. Infections lead to microcirculatory disorders, which occur more frequently in the skin and subcutaneous fat tissue, exacerbating circulatory disorders. To account for this effect in the present formula, pTOI was multiplied by the thickness of the subcutaneous fat layer.

Comparing the obtained parameters of peripheral(-muscle) oxygenation with previous published values [23,24], pTOI values from the present study in the entire cohort (72.6 ± 5.5%) can align with published normal values within the first six hours after birth in term (71.5 [68.2–74.8] %) [24] and moderate-to-late-preterm neonates (69.7 [67.6–75.4] %) [23].

Comparing the present pTOI values (74.5 [69.0; 80.2] %) in neonates with elevated CRP levels ≥ 20 mg/L with pTOI values (68.9 ± 6.6%) of neonates with elevated CRP levels in the study by Pichler et al. [17], a clear difference can be noted, with lower pTOI values in the study by Pichler et al. [17]. A potential explanation for this discrepancy might be the gestational and the postnatal age, as the literature describes a significant negative correlation of postnatal age and a positive correlation of gestational age with pTOI [18]. Mean gestational age in the present study was higher compared to the cohort described by Pichler et al. (mean gestational age 37.7 ± 2.9 weeks) [17]. Peripheral(-muscle) NIRS measurements in the present cohort were performed within the first six hours after birth compared to 41 ± 25 h in the study by Pichler et al. [17]. We assume that the higher gestational age outweighs the effect of postnatal age, resulting in higher pTOI values in our present cohort compared to those reported by Pichler et al. [17].

A behaviour comparable, however reciprocal, to pTOI is shown by pFTOE, which represents the balance between oxygen consumption and oxygen delivery to the tissue. In our present study, pFTOE in the entire cohort is similar to the established pFTOE normal values within the first six hours after birth [24]. However, again, a difference in pFTOE can be observed in neonates of the present study with elevated CRP levels ≥ 20 mg/L (pFTOE = 0.215 [0.119; 0.288]) compared to the established normal values in term (0.264 (0.229–0.300) and preterm neonates 0.229 (0.213–0.246) measured within the same time period after birth. This observation contradicts our expectations, as we would have expected an increase in pFTOE in case of microcirculation disturbances. A potential explanation for the these observations might be provided by Hoeller et al. [32], who described lower pFTOE values and a reduced ability to detect impaired microcirculation compared to the peripheral(-muscle) fractional oxygen extraction (pFOE) obtained through peripheral(-muscle) NIRS measurements combined with the venous occlusion method.

The POP-Score should not be interpreted as a diagnostic or predictive tool. Rather, it represents a preliminary conceptual approach that requires formal model development, validation, and testing in adequately powered studies.

### Limitation

The major limitation of the present study is the small number (n = 6) of included neonates with a CRP level ≥ 20 mg/L, resulting in an underpowered analysis and unreliable estimates of diagnostic accuracy. Due to the matching by gestational age, the overall number of included neonates was further reduced. Although we were able to describe both a sensitivity of 100% and specificity of 87% for exploring association with elevated CRP levels ≥ 20 mg/L, the limited sample size poses a significant constraint. The present study was not designed to evaluate predictive performance, and any apparent predictive capability of the POP-Score may reflect overfitting to this specific dataset.

The POP-Score was not derived using multivariable modelling, and no ROC analysis, calibration assessment, or internal validation was performed. Therefore, its robustness and generalizability remain unknown. The current formulation reflects a clinically motivated combination of parameters rather than a statistically optimized model. Validation in independent and adequately powered cohorts is required. A critical aspect of the present study is the use of elevated CRP levels as a surrogate marker for infection. Although CRP is widely used in clinical practice as an indicator of neonatal infection, it is a non-specific inflammatory marker and may be elevated in various non-infectious conditions such as birth stress, hypoxia, or perinatal asphyxia. Furthermore, the temporal dynamics of CRP, including its delayed increase within the first 10–12 h after onset of inflammation, may limit its accuracy in reflecting early infectious processes. Therefore, elevated CRP levels do not necessarily correspond to microbiologically confirmed infection or sepsis. Despite these limitations, CRP remains a clinically relevant and routinely used parameter in neonatal care, particularly in situations where blood cultures are not feasible or yield inconclusive results. In this context, the POP-Score should be interpreted as a tool to be associated with inflammatory response rather than confirmed infection. Future studies should aim to validate the POP-Score against clinically confirmed infection, including positive blood cultures and standardized sepsis definitions.

A further limitation is that we did not assess whether the POP-Score improves clinical decision making or reduces unnecessary antibiotic use. Additionally, no comparison with established biomarkers such as CRP alone or with clinical scoring systems was performed. Therefore, the added clinical value of the POP-Score beyond current standard practice remains unclear.

Peripheral(-muscle) NIRS measurements are known to be influenced by several factors, including probe positioning, motion artefacts, and tissue composition. Although a standardized measurement protocol was applied in the present study, including repeated measurements and averaging, residual variability cannot be excluded. In particular, subcutaneous fat layer thickness and local tissue characteristics may affect signal quality and comparability between patients. While we attempted to account for this by incorporating subcutaneous fat thickness into the POP-Score, measurement variability remains a potential limitation.

Furthermore, since we included relevant factors influencing peripheral(-muscle) oxygenation (subcutaneous fat layer thickness, SpO_2_, HR, SABP), we assume that our provided POP-Score has the potential to serve as a conceptual approach in detecting early stages of infections. These findings should be considered hypothesis-generating.

## 5. Conclusions

In this study, we introduce the POP-Score with a cut-off value of 1.00 for exploring association with elevated CRP levels ≥ 20 mg/L in neonates with a birth weight ≥ 2000 g and signs of respiratory distress within the first six hours after birth. This cut-off value of 1.00 for the POP-Score showed a sign of association with elevated CRP levels ≥ 20 mg/L and therefore may support the early identification of neonates at risk of infection/inflammatory conditions. The combination of different parameters, as applied in our POP-Score, may help to reflect early physiological changes associated with inflammation in neonates when other routine monitoring parameters are still within normal ranges. However, the POP-Score should be considered a hypothesis-generating tool rather than a diagnostic marker, and its findings should be interpreted with caution given the limited sample size. No inference regarding clinical decision making should be drawn from the present results.

To address the limitations of this study, a prospective study (POP-Score Trial, clinicaltrials.gov NCT07109856) is currently ongoing to evaluate the performance of the POP-Score in a larger cohort. This study is designed to assess its predictive value in comparison to established biomarkers, its added value to clinical decision making, and its potential to reduce unnecessary antibiotic exposure in neonates.

## Figures and Tables

**Table 1:** Demographic data, routine monitoring parameters, and peripheral(-muscle) near-infrared spectroscopy parameters of 30 included term and late-preterm neonates in the “CRP ≥ 20 mg/L group” and “CRP < 20 mg/L group”. Values are displayed as median (minimum; maximum) and n (%).

	CRP ≥ 20 mg/L Group n = 6	CRP < 20 mg/L Group n = 24	*p*-Value
Female sex, n (%)	1 (16.7%)	11 (45.8%)	0.358
Apgar 1	9 (8; 9)	9 (4; 9)	0.723
Apgar 5	9 (8; 10)	9 (8; 10)	0.978
Apgar 10	10 (9; 10)	9 (9; 10)	0.717
Umbilical cord artery pH	7.32 (7.31; 7.39)	7.30 (7.24; 7.42)	1.000
Postnatal age [min]	147 (114; 343)	205 (75; 354)	0.178
Subcutaneous fat layer thickness [cm]	0.65 (0.46; 0.74)	0.49 (0.25; 0.96)	0.020
Diameter of the forearm [cm]	3.6 (3.2; 4.1)	3.3 (2.6; 3.6)	0.102
Body temperature [°C]	37.4 (36.7; 38.1)	37.2 (36.9; 37.7)	0.727
Arterial oxygen saturation [%]	95 (91; 96)	96 (91; 99)	0.500
Heart rate [bpm]	158 (144; 163)	132 (114; 156)	0.017
Systolic blood pressure [mmHg]	59 (54; 69)	58 (50; 65)	0.586
Diastolic blood pressure [mmHg]	36 (32; 42)	33 (28; 44)	0.938
Mean arterial blood pressure [mmHg]	45 (40; 51)	41 (36; 49)	0.876
Leukocyte counts [/µL]	19,825 (11,300; 25,160)	16,950 (11,680; 24,700)	0.678
IT ratio	0.03 (0.01; 0.23)	0.02 (0.00; 0.10)	0.327
pTOI [%]	74.5 (69.0; 80.2)	73.2 (68.8; 80.2)	0.421
pFTOE	0.215 (0.119; 0.288)	0.235 (0.164; 0.283)	0.378
pH	7.37 (7.30; 7.41)	7.31 (7.23; 7.41)	0.169
pCO_2_ [mmHg]	42.0 (35.0; 58.4)	51.1 (40.1; 67.1)	0.325
HCO_3_^−^ [mmol/L]	22.8 (22.7; 24.7)	23.1 (21.4; 26.3)	0.568
Base deficient [mmol/L]	−1.0 (−2.3; 2.7)	−0.5 (−2.8; 2.5)	0.659
Lactate [mmol/L]	2.0 (1.5; 3.2)	1.5 (0.7; 7.0)	0.138

Abbreviations: CRP = C-reactive protein, HCO_3_^−^ = hydrogen carbonate, IT ratio = immature-total neutrophil ratio, pCO_2_ = partial pressure of carbon dioxide, pFTOE = peripheral(-muscle) fractional tissue oxygen extraction, pTOI = peripheral(-muscle) tissue oxygenation.

## Data Availability

The data presented in this study are available on request from the corresponding author.

## Abbreviations

The following abbreviations are used in this manuscript:

SpO2	arterial oxygen saturation
crSO2	cerebral oxygen saturation
CRP	C-reactive protein
DABP	diastolic blood pressure
HR	heart rate
IT ratio	immature total neutrophil ratio
MABP	mean arterial blood pressure
NIRS	near-infrared spectroscopy
NICUs	neonatal intensive care units
pFTOE	peripheral(-muscle) fractional tissue oxygen extraction
POP-Score	peripheral(-muscle) oxygenation and perfusion Score
pTOI	peripheral(-muscle) tissue oxygenation
SABP	systolic blood pressure
